# The factors associated with cognitive function among community-dwelling older adults in Taiwan

**DOI:** 10.1186/s12877-023-03806-4

**Published:** 2023-03-02

**Authors:** You-Chen Mary Lor, Meng-Ting Tsou, Li-Wei Tsai, Szu-Ying Tsai

**Affiliations:** 1 Department of Family Medicine, Hsinchu MacKay Memorial Hospital, No. 690, Section 2, Guangfu Road, East District, Hsinchu, 300 Taiwan; 2 Department of Family Medicine, MacKay Memorial Hospital, Taipei, Taiwan; 3 Department of Nursing and Management, MacKay Junior College of Medicine, New Taipei City, Taiwan; 4 Department of Surgical Oncology, National Taiwan University Cancer Center, Taipei, Taiwan; 5 Department of Surgery, National Taiwan University Hospital and National Taiwan University College of Medicine, Taipei, Taiwan

**Keywords:** Aged, Cognition, Community-dwelling older adults, Diabetes mellitus, Hyperlipidemia

## Abstract

**Background:**

This research aimed to investigate the associations of anthropometric measurements, physiological parameters, chronic disease comorbidities, and social and lifestyle factors with cognitive function amongst community-dwelling older adults in Taiwan.

**Methods:**

This was an observational, cross-sectional study involving 4,578 participants at least 65 years old, recruited between January 2008 and December 2018 from the Annual Geriatric Health Examinations Program. Cognitive function was assessed using the short portable mental state questionnaire (SPMSQ). Multivariable logistic regression was done to analyze the factors associated with cognitive impairment.

**Results:**

Among the 4,578 participants, 103 people (2.3%) with cognitive impairment were identified. Associated factors were age (odds ratio (OR) = 1.16, 95% confidence interval (CI) = 1.13,1.20), male gender (OR = 0.39, 95% CI = 0.21,0.72), diabetes mellitus (DM) (OR = 1.70, 95% CI = 1.03, 2.82), hyperlipidemia (OR = 0.47, 95% CI = 0.25, 0.89), exercise (OR = 0.44, 95% CI = 0.34, 0.56), albumin (OR = 0.37, 95% CI = 0.15, 0.88), and high-density lipoprotein (HDL) (OR = 0.98, 95% CI = 0.97, 1.00). Whereas waistline, alcohol intake in recent six months, and hemoglobin was not significantly associated with cognitive impairment (all *p* > 0.05).

**Conclusions:**

Our findings suggested that people with older age and a history of DM had a higher risk of cognitive impairment. Male gender, a history of hyperlipidemia, exercise, a high albumin level, and a high HDL level seemed to be associated with a lower risk of cognitive impairment amongst older adults.

**Supplementary Information:**

The online version contains supplementary material available at 10.1186/s12877-023-03806-4.

## Background

The trend towards a rapidly aging society is a manifest global issue that brings subsequent health challenges worldwide. Regardless of geographic location, developed countries and developing countries alike will both face expected increases in health care demand and related socioeconomic burdens [1]. In 2015, there were 617.1 million people (9 percent of the world population) aged 65 and older. By 2030, this population will increase to approximately 1 billion, equivalent to 12 percent of the predicted total world population. By 2050, this older population is estimated to be 1.6 billion or 17 percent of the entire global population [2]. According to a report from the US Economics and Statistics Administration, Department of Commerce this rapid growth of an aged society is also being observed in Asia, with an estimation of Asia’s older population almost tripling in size from 341.4 million in 2015 to 975.3 million in 2050 [2].

An aged society faces numerous health issues. Advances in human civilization have increased human life expectancy, but the subsequent health care problems that accompany ageing will cause a heavy care burden [3]. For example, cognitive decline is a well-recognized problem in older adults [3, 4]. When mild cognitive impairment in older adults progresses to dementia, it typically causes disability and is related to a higher mortality risk [5–7]. Cognitive dysfunction is associated with a poor quality of life in older adults [8]. Thus, finding ways to decelerate or even stop cognitive decline has become an important issue nowadays. In line with the recommendation from the United Nations “Decade of Healthy Ageing (2021–2030)” report, taking action to prevent cognitive function decline is important in achieving successful aging [9].

There is numerous evidence that age plays a crucial role in the cognitive function decline process [10, 11]. However, there are still some modifiable factors that are associated with impaired cognitive function [12]. Studies have linked lower cognitive performance and risk of dementia with diabetic individuals [12–15]. Obesity, on the other hand, has been discussed with controversy. Obesity has been reported to be associated with cognitive decline in some studies [16], while others propose the opposite point of view [17, 18]. Additionally, a history of myocardial infarction [17], hypertension [19], stroke, and depression [19] have all been found to be independently associated with a higher cognitive impairment prevalence among different population groups. Lifestyle and nutrition factors discussed in some studies – such as the consumption of different diets, variety of fruit or vegetable intake, or beverage consumption have shown divergent results [17, 20]. Physical activity, as compared to no exercise, was associated with a lower risk of cognitive impairment, Alzheimer’s disease, and dementia of any type [21–25]. Other independent risk factors for cognitive impairment, like tobacco and alcohol use, have also been studied [26].

The WHO defines [27] a hyper-aged society as a society where the aged population accounts for more than 20% of the total population. In the report of the “Population Projections for R.O.C. (Taiwan): 2016–2061” published by the National Development Council, Taiwan is expected to become a hyper-aged society by 2026 [27]. This aging rate is faster than the rates of other developed countries. Thus, prevention of cognitive impairment is an important health issue, especially in the rapidly ageing societies in Asia. However, studies examining the modifiable factors associated with cognitive decline in community-dwelling and relatively healthy Taiwanese geriatrics are still lacking [22, 28, 29]. Therefore, this study aimed to investigate the factors associated with cognitive function in community-dwelling Taiwanese older adults patients aged 65 or older.

## Methods

### Study population

This is an observational, cross-sectional study. The participants were recruited between January 2008 and December 2018 from the Annual Geriatric Health Examinations Program at MacKay Memorial Hospital, which is a tertiary medical center with 1,981 hospital beds in northern Taiwan spread across two branches located in Taipei and New Taipei. Since 2008, public health bureaus and hospitals in each local jurisdiction have participated in the Annual Geriatric Health Examinations Program, which is sponsored by Taiwan’s Health Promotion Administration, Ministry of Health and Welfare [30]. Any citizen aged 65 or older is eligible to take part. Components of the annual health examination include anthropometric measurements, sociodemographic data collection, cognitive function examination, depression screening, hemogram and biochemistry lab data, physical examination, and a health consultation. Different city and county public health bureaus may also provide additional examinations such as abdominal ultrasounds, resting electrocardiography, spine X ray, chest X ray, stool analysis, cancer biomarkers, or urine analysis. In 2018, the attending rate of the annual geriatric physical health examination program was 29.34% among all Taiwanese seniors, and 20.79% and 24.73 among seniors from Taipei and New Taipei, respectively [31].

**Fig. 1:**
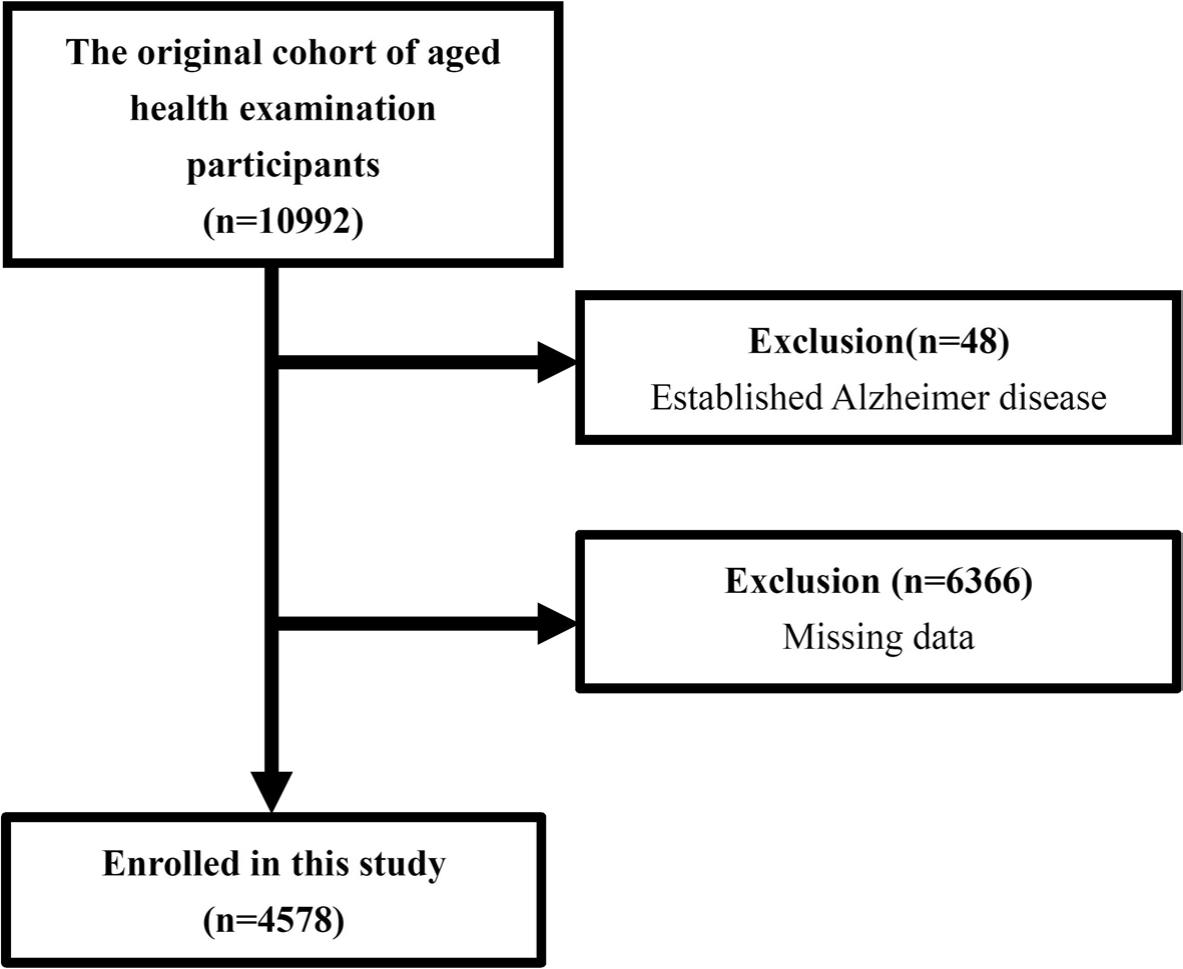
The selection algorithm of study participants

Our study participants initially included 10,992 people aged 65 or older. However, 48 participants found to have Alzheimer’s disease through history taking or current medication use were excluded. Participants with any missing data were also excluded, thus resulting in a total of 4,578 older adults enrolled in the study (Fig. 1). The study protocol was evaluated and approved by the MacKay Memorial Hospital Institutional Review Board Approval of Clinical Trial (project research number 18MMHIS137).

### Data collection

All participants fasted for at least eight hours before their blood test to minimize possible confounding factors on serum fasting glucose and lipid profiles. Hemogram and biochemical lab data were analyzed through standard protocol procedures by the laboratory department in the hospital. Sociodemographic information was obtained through face-to-face interviews by trained nurses using a uniform questionnaire. The participants’ age was calculated by subtracting the date of birth from the date of the examination. Comorbidities, including a clinical history of hypertension, diabetes mellitus (DM), hyperlipidemia, cardiovascular diseases (CVD), depression, osteoporosis, and hyperthyroidism, were established by self-reporting on pre-existing diagnosis and medication. CVD was defined as coronary heart disease with or without stent, ischemic heart disease, myocardial infarction, angina pectoris, and stroke. Structural heart diseases and congenital heart diseases were not included in the cardiovascular disease definition in our study. An exercise habit within the past six months was defined as exercise at least three times a week, lasting at least 20 min each time. Smoking, alcohol drinking, and betel nut chewing status were evaluated and the participant would be allocated to the “yes” group if he or she had used one of these substances one or more times within the past six months.

Trained nurses measured and recorded all anthropometric measurements. Both body height and body weight were obtained from an automatic scale. Body mass index (BMI) was calculated using the following equation: body weight (kilogram) divided by body height (meter) squared. Waist circumference (WC) was measured at the middle point between the last rib margin and the iliac crest. To obtain an office blood pressure, the blood pressure was measured at least twice using a sphygmomanometer on the participant’s right arm after a 10-min rest. The average blood pressure was calculated from the measurements and adopted in this study.

### Assessment of cognitive function

The Short Portable Mental Status Questionnaire (SPMSQ) was developed by Pfeiffer in 1975 to rapidly screen patients with organic brain syndromes [32]. According to Erkinjuntti et al., using the cut-off point (number of errors accepted) of three errors, the sensitivity of the test was 86.2% and the specificity 99.0% among medical inpatients and 66.7% and 100%, respectively, among community residents [33]. One study indicated that when using SPMSQ to assess the intellectual functioning of older adults, the two-group model (intact/mildly impaired and moderately/severely impaired) permitted significant discrimination [34]. Many studies have shown that SPMSQ is a useful and efficient tool to identify cognitive impairment [7, 33, 35, 36]. Thus, we chose to administer the SPMSQ to screen for cognitive impairment in our study. It has been adopted and validated for use in Taiwanese adults [22, 30]. It contains 10 questions, and was administered face-to-face in Mandarin or Taiwanese according to the patient’s native or preferred language by the interviewers in our study. The interviewers were trained nurses. The questions as listed below were: (1) What is the date, month, and year? (2) What is the day of the week? (3) What is the name of this place? (4) What is your phone number? (5) How old are you? (6) When were you born? (7) Who is the current president? (8) Who was the president before him or her? (9) What was your mother’s maiden name? (10) Can you count backward from 20 by threes [32]? One mark was given for each wrong answer. A score equal to or fewer than 2 stood for intact cognitive performance while a score of 3 or more represented impaired cognitive function [32, 34]. The higher the score in SPMSQ, the worse the cognitive function. In our study, we categorized participants with SPMSQ scores equal to or greater than 3 into the cognitive impairment group and those with SPMSQ scores equal to or fewer than 2 into the normal cognitive function group.

### Statistical analysis and outcome measurement

The quantitative data was analyzed with the IBM SPSS 20.0 version. The two-tailed significance level was *p* < 0.05. Among the demographic data, continuous variables were presented as a mean ± SD and categorical variables were shown as a frequency and percentage. Comparisons were made between the basic demographic data in the normal cognitive function and the cognitive impairment group using the independent T-test for continuous variables and Chi-square for categorical variables. Multivariate logistic regression comprising variables with statistical significance in Table 2 were used to determine the factors associated with cognitive impairment.

## Results

Our study included 4,578 participants aged 65 years or older (Fig. 1). The average age was 73.5 ± 5.8 years old. Male participants accounted for 27.1% of the total number of participants. The average BMI was 24.4 ± 3.4 (kg/m^2^), and the WC was 83.2 ± 10.0 (cm). The mean blood pressure was systolic blood pressure (SBP) = 136.4 ± 19.9(mmHg) and diastolic blood pressure (DBP) = 71.1 ± 11.1(mmHg). Other baseline demographic data, including chronic disease history, social habits, and biochemistry lab data, are summarized in Table 1. The average SPMSQ score was 0.2 ± 0.9 (Table 1). The distribution of participants’ SPMSQ scores is shown in Table S1.

**Table 1:** Demographic characteristics of study subjects

	**All participants(*N* = 4578)**
Age (years)	73.5 ± 5.8
Gender (male)	1240 (27.1%)
Body height (cm)	155.2 ± 7.6
Body weight (kg)	58.9 ± 9.8
Body mass index (kg/m^2^)	24.4 ± 3.4
Systolic blood pressure (mmHg)	136.4 ± 19.9
Diastolic blood pressure (mmHg)	71.1 ± 11.1
Pulse rate (bpm)	73.9 ± 11.6
Waist circumference (cm)	83.2 ± 10.0
Hypertension (yes)	2300 (50.2%)
Diabetes Mellitus (yes)	698 (15.2%)
Hyperlipidemia (yes)	1007 (22.0%)
Depression (yes)	30(0.7%)
Cardiovascular disease (yes)	787(17.2%)
Osteoporosis (yes)	58 (1.3%)
Hyperthyroidism (yes)	38 (0.8%)
Smoking in 6 months(yes)	157 (3.4%)
Alcohol in 6 months(yes)	524 (11.4%)
Betel nut in 6 months(yes)	9 (0.2%)
Exercise in 6 months(yes)	2820 (61.6%)
AC sugar (mg/dL)	106.6 ± 23.2
Total protein (g/dL)	7.3 ± 0.5
Albumin (g/dL)	4.2 ± 0.4
GOT (U/L)	25.5 ± 14.5
GPT (U/L)	22.4 ± 16.0
Creatinine (mg/dL)	1.0 ± 0.4
Total cholesterol (mg/dL)	198.7 ± 34.5
Triglyceride (mg/dL)	118.3 ± 67.1
High density lipoprotein (mg/dL)	58.2 ± 16.1
Uric acid (mg/dL)	5.6 ± 1.4
Hemoglobin (g/dL)	13.2 ± 1.3
SPMSQ score (scores)	0.2 ± 0.9

The continuous variables were shown as mean ± SD; the categorical variables were shown as percentage

Abbreviations: *SPMSQ* Short portable mental status questionnaire

Overall, participants without cognitive impairment (SPMSQ < 3) made up 97.8% of the whole study group, with the majority of the total participants (88.6%) scoring a perfect score of 0 (Table S1). In total, there were 103 participants with cognitive impairment, comprising 2.3% of the study group. Table 2 presents the differences noted between the normal cognitive function group (SPMSQ < 3) and the cognitive impairment group (SPMSQ≧3). There was a statistically significant difference in age between the group with cognitive impairment, at 79.7 ± 7.5 years old on average, compared with the normal cognitive function group, at 73.3 ± 5.7 years old. Participants in the cognitive impairment group were typically older females with a larger waist circumference. They were more likely to have DM, and less likely to have hyperlipidemia, exercise regularly, or drink alcohol. Lower levels of albumin, high-density lipoprotein (HDL) and hemoglobin were also noted in the cognitive impairment group. Although there were discrepancies in body height and body weight between the two groups, the calculated BMI showed no significant difference (Table 2).

**Table 2:** Comparisons of cognitive impairmentgroup (SPMSQ≧3) and normal cognitive function group (SPMSQ < 3)

	**SPMSQ≧3 (*N* = 103)**	**SPMSQ < 3 (*N* = 4475)**	***P* value**
Age (years)	79.7 ± 7.5	73.3 ± 5.7	**< 0.001**
Gender (male)	15 (14.6%)	1225 (27.4%)	**0.004**
Body height (cm)	150 ± 7.6	155.3 ± 7.6	**< 0.001**
Body weight (kg)	54.4 ± 9.5	59.0 ± 9.8	**< 0.001**
Body mass index (kg/m^2^)	24.2 ± 3.9	24.4 ± 3.4	0.546
Systolic blood pressure (mmHg)	138.8 ± 23.4	136.4 ± 19.8	0.298
Diastolic blood pressure (mmHg)	69.3 ± 12.3	71.2 ± 11.1	0.093
Pulse rate (bpm)	76.1 ± 13.3	73.9 ± 11.5	0.098
Waist circumference (cm)	85.6 ± 10.5	83.1 ± 10.0	**0.014**
Hypertension (yes)	59 (57.3%)	2241 (50.1%)	0.148
Diabetes Mellitus (yes)	26 (25.5%)	672 (15%)	**0.004**
Hyperlipidemia (yes)	12 (11.7%)	995 (22.2%)	**0.010**
Depression (yes)	0 (0%)	30 (0.7%)	0.404
Cardiovascular disease (yes)	24 (23.3%)	763 (17.1%)	0.096
Osteoporosis (yes)	2 (1.9%)	56 (1.3%)	0.536
Hyperthyroidism (yes)	0 (0%)	38 (0.8%)	0.348
Smoking in 6 months(yes)	5 (4.9%)	152 (3.4%)	0.422
Alcohol in 6 months(yes)	5 (4.9%)	519 (11.6%)	**0.034**
Betel nut in 6 months(yes)	1 (1.0%)	8 (0.2%)	0.073
Exercise in 6 months(yes)	31 (30.1%)	2789 (62.3%)	**< 0.001**
AC sugar (mg/dL)	110.2 ± 24.9	106.5 ± 23.2	0.138
Total protein (g/dL)	7.3 ± 0.6	7.3 ± 0.5	0.529
Albumin (g/dL)	4.0 ± 0.2	4.2 ± 0.4	**< 0.001**
GOT (U/L)	29.6 ± 35.3	25.5 ± 13.6	0.240
GPT (U/L)	27.5 ± 48.8	22.3 ± 14.3	0.285
Creatinine (mg/dL)	1.0 ± 0.2	1.0 ± 0.4	0.448
Total cholesterol (mg/dL)	194.9 ± 32.9	198.8 ± 34.5	0.247
Triglyceride (mg/dL)	127.5 ± 54.7	118.1 ± 67.3	0.161
High density lipoprotein (mg/dL)	53.8 ± 15.4	58.3 ± 16.1	**0.005**
Uric acid (mg/dL)	5.5 ± 1.8	5.6 ± 1.4	0.881
Hemoglobin (g/dL)	12.8 ± 1.4	13.2 ± 1.3	**0.006**

The continuous variables were shown as mean ± SD; the categorical variables were shown as percentage. Using chi-squared and t-test; Statistical significance was defined as *P* < 0.05

Abbreviations: *SPMSQ* Short portable mental status questionnaire

A multivariate logistic regression analysis was done on the variables mentioned above that presented significant differences between the two groups (Table 3). Age (odds ratio (OR) = 1.16, 95% confidence interval (CI) = 1.13, 1.20) and DM (OR = 1.70, 95% CI = 1.03, 2.82) were found to be positively associated with cognitive impairment. In contrast, male gender (OR = 0.39, 95% CI = 0.21, 0.72), hyperlipidemia (OR = 0.47, 95% CI = 0.25, 0.89), exercise (OR = 0.44, 95% CI = 0.34, 0.56), albumin (OR = 0.37, 95% CI = 0.15, 0.88), and HDL level (OR = 0.98, 95% CI = 0.97, 1.00) were negatively associated with the cognitive impairment group. The former associations of waistline, recent alcohol intake, and hemoglobin level as stated previously in Table 2 did not remain significantly different after multivariate adjustment (Table 3).

**Table 3:** Multivariate logistic regression of factors associated with cognitive impairment(SPMSQ≧3)

	**Odds Ratio Cognitive decline (SPMSQ≧3)**	***P* value**
Age (years)	1.16 (1.13, 1.20)^*^	< 0.001
Gender (male)	0.39 (0.21, 0.72)^*^	0.003
Waist circumference (cm)	1.01 (0.99, 1.03)	0.440
Diabetes Mellitus (yes)	1.70 (1.03, 2.82)^*^	0.038
Hyperlipidemia (yes)	0.47 (0.25, 0.89)^*^	0.020
Alcohol in 6 months(yes)	0.80 (0.31, 2.08)	0.646
Exercise in 6 months(yes)	0.44 (0.34, 0.56)^*^	< 0.001
Albumin (g/dL)	0.37 (0.15, 0.88)^*^	0.024
High density lipoprotein (mg/dL)	0.98 (0.97, 1.00)^*^	0.024
Hemoglobin (g/dL)	1.07 (0.91, 1.25)	0.448

Odds ratio (OR) and 95% confidence intervals (CIs) are shown after multivariate logistic regression

^*^Data are statistically significant (*p* < 0.05)

Abbreviations: *SPMSQ* Short portable mental status questionnaire

In order to obtain a broader perspective, we did a further analysis by including in our data the samples that were originally excluded due to previous missing values. The characteristics of the total 10,944 participants are summarized in Table S2. The comparison of the two groups (the normal cognitive function and the cognitive impairment group) is shown in Table S3. Multivariate logistic regression analysis of the factors associated with cognitive impairment is shown in Table S4. Exercise in six months, higher albumin and HDL levels were significantly associated with a lower risk of cognitive impairment. Increasing age was found to be significantly associated with cognitive impairment. Although not significantly found, male gender, DM and hyperlipidemia also presented a higher risk of cognitive impairment. Overall, the results of the multivariate logistic regression in Table S4 were comparable to our main results in Table 3. Both seem to have render similar conclusions.

## Discussion

Our study is an observational, cross-sectional study examining the modifiable factors associated with cognitive impairment in Taiwan’s community-dwelling older adults. The factors we observed to be associated with increased risk of cognitive impairment were age and diabetes mellitus. Male gender, hyperlipidemia, exercise, albumin level, and HDL level were related to a lower risk of cognitive impairment.

A prevalence of 2.3% older adults with cognitive impairment was observed in our study, which was less than in the other studies. The prevalence of cognitive impairment was 22.2% in a previous Taiwanese study [37]. In Asia, the prevalence of cognitive impairment ranges from 13.29% to 21.5% [17, 38]. This disparate finding could be due to our study participants being recruited from the Annual Geriatric Health Examinations Program, which on average comprises only 20 to 30% of all Taipei and New Taipei seniors, with variation for each year, thus there still remained a residual selection bias. Taipei and New Taipei both represent a highly urbanized area. Previous studies analyzing the urban–rural differences in the prevalence of mild cognitive impairment (MCI) of older adults in Taiwan have suggested a lower prevalence of MCI in the urban community than in the rural one [29, 39]. In addition, seniors that voluntarily partake in the yearly geriatric health exam tend to have better physical and mental health, including autonomy in activities of daily living, higher social participation, and little or no disability. However, even with the small proportion of participants that were found to be have cognitive impairment, we were still able to find significant differences in characteristics between the SPMSQ≧ 3 and SPMSQ < 3 groups.

We observed a higher prevalence of cognitive impairment in females than in males, which is consistent with a previous cross-sectional study done exploring age and sex-specific prevalence among older adults with mild cognitive impairment [23]. Our study also demonstrated an association between cognitive impairment and ageing. This is consistent with previous studies that have shown ageing to be a key risk factor in cognitive decline [10, 11]. A history of diabetes mellitus was also shown to have a correlation with our cognitive impairment group. Diabetes mellitus as a risk factor for cognitive impairment has been studied previously [14, 15]. Suggested mechanisms underlying this relationship include neurotoxic effects on brain cells, increased production of Reactive Oxygen Species (ROS), and accelerated brain microangiopathy development when hyperglycemia is present [40, 41].

In this study, presence of a history of hyperlipidemia, regular exercise, higher albumin level, and higher HDL level were related to a lower risk of cognitive impairment. Hyperlipidemia in the role of cognitive decline has been up to debate, as previous studies have shown discrepant results. Some studies have shown that elevations in total cholesterol and low-density lipoprotein cholesterol (LDL) were related to decreased cognitive performance [42, 43] and mild cognitive impairment [44]. However, other studies had null results and did not find that total cholesterol [45] or a history of hyperlipidemia [46] was linked to cognitive decline. Our data revealed that a history of hyperlipidemia and higher levels of HDL were associated with the normal cognitive function group. This is in line with previous research that has shown low HDL to be detrimental to cognition [47, 48]. Cholesterol dysregulation has been implicated in the development of neurodegenerative diseases, such as Alzheimer’s disease, Parkinson’s disease and Huntington’s disease. An animal study done on Huntington’s disease mice found decreased cholesterol synthesis in the striatum of the brain. Injection of cholesterol directly into the striatum ameliorated some motor symptoms and prevented cognitive decline [49]. Cholesterol is crucial in ensuring normal brain function, as it is an important component of the cell membrane [50]. Increase in cholesterol over time was associated with better cognition in a longitudinal study [51]. A previous study suggested that a lower total cholesterol could be used as a marker to predict cognitive decline in older adults [52]. Could a higher total cholesterol be an indicator of non-frailty and thus be protective for cognitive function? Could a history of hyperlipidemia in the normal cognitive function group suggest that lipid lowering agents play a role in improving cognition? Prospective cohort studies in the past examining the role of lipid lowering agents in cognitive decline did not find that the medication was preventive against cognitive decline or dementia [53, 54]. Thus, the precise role of hyperlipidemia on cognitive function needs to be explored further.

Our participants in the cognitive impairment group were less likely to be physically active. Overall, 62.3% of the participants without cognitive impairment compared to only 30.1% of the cognitive impairment group had an exercise habit within the past six months. This is consistent with previous studies that have reported a positive association between exercise and cognitive function [23, 24]. A meta-analysis of 15 studies concluded that physical activity significantly protected against cognitive decline [25]. Current and consistent exercise habits among Taiwanese seniors led to better cognitive performance on the SPMSQ over the course of an eight-year follow-up study [22].

Our results indicated that participants with a higher albumin level had a lower risk of cognitive impairment. Lower albumin levels have been reported to be correlated with poor cognitive performance in older adults [55–57]. In a previous study done in Japan [58], a positive association between the serum albumin/globulin ratio (A/G ratio) and cognitive function was found in 70-year-old and 80-year-old participants. Similar to our study, the Asian study participants also had normal albumin levels. Since albumin is seen as a marker for nutritional status and inflammation, a low albumin level may indicate malnutrition, chronic hepatitis, nephrotic syndrome or an inflammation status. Thus, higher albumin levels may be associated with healthier individuals and possibly having better cognitive function as well.

Evidence exploring the relationship between waist circumference and cognitive impairment has had conflicting results. Some studies have reported that an increase in waist circumference was associated with cognitive decline risk [59, 60]. However other studies have shown that greater waist circumference was associated with slower cognitive decline [61, 62] or produced null results similar to our own [38]. Further studies are warranted before a consensus is reached regarding the effect of waist circumference on cognitive performance. We also did not observe any significant association between alcohol drinking or hemoglobin levels with cognitive impairment. In addition, SBP, DBP and a clinical history of hypertension were not found to significantly different between the cognitive impairment group and normal cognitive function group. Whereas in a previous study, high blood pressure, hypertension, uncontrolled blood pressure was associated with poorer cognitive function when compared with those whom had normal blood pressure in participants aged 70 and older [63]. However, this association was not shown in participants aged 60 to 69. In a study done in Japan, high SBP was found to be significantly correlated with reduced cognitive functioning in 70-year-old participants, but not in participants aged 80 years old [64]. Both of these studies suggest that high blood pressure may be a risk factor for cognitive decline in subjects around 70-year-old, but the results were not consistent amongst other age groups. Blood pressure readings may be affected by the clinic environment, emotional stress and the well-being of the person. Well-controlled hypertension relies on good compliance of antihypertensive drugs, smoking abstinence, regular exercise, and a well-balanced diet. There may have been some confounding factors in play that we did not take in account of and thus, our study did not find any association between hypertension and cognitive function. More studies are needed to verify the relationship between blood pressure and cognition.

Our study focusing on Taiwanese older adults aimed to find out the modifiable risk factors of cognitive impairment to mitigate the subsequent care burden of a rapidly-aging society. Strengths of our study include having a relatively large number of participants and the use of a rigorous and standardized protocol for data collection. The data collected for our study from the Annual Geriatric Health Examinations Program was methodically done with trained nurses and doctors gathering the participants’ information and carrying out the anthropometric measurements. Nevertheless, some limitations should be considered. First, although the SPMSQ is a tool used in cognitive function decline screening, it cannot be used to diagnose dementia. The participants with SPMSQ scores ≧ 3 in our study were identified as having cognitive impairment, but as for its use in screening for dementia, this remains uncertain. Second, other potential risk factors like educational level, income level, diet habits, and history of other comorbidities were not analyzed in this study. Furthermore, participants’ use of anti-hypertensive, anti-hyperglycemic, and lipid-lowering agents was also not examined. Although we included exercise, smoking, and alcohol consumption in our study, more detailed exploration of the type of exercise and its intensity as well as the type and amount of alcohol and cigarette consumption could be useful for future studies and analyses. Finally, only a small percentage (2.3%) of our participants were found to have a score of SPMSQ≧3. This large disparity in sample size between the SPMSQ≧ 3 and SPMSQ < 3 groups could be due to that most community-dwelling older adults who voluntarily participate in the walk-in Annual Geriatric Health Examinations Program usually have little or no disability and are relatively healthy. This may have introduced bias in our results and limits the generalizability of our findings. Perhaps, a different cognitive screening tool with a wider scale distribution such as the Montreal Cognitive Assessment (MoCA) test could be considered for future studies. Further studies are still required to identify the risk factors pertaining to cognitive impairment.

## Conclusions

In conclusion, in this cross-sectional study of community-dwelling older individuals in Taiwan, older age and a history of diabetes mellitus were associated with an increased risk of cognitive impairment. Therefore, control of one’s blood glucose may have beneficial effects on cognitive function. Alternatively, being a male, having a history of hyperlipidemia, regular exercise, and higher albumin and HDL levels were related to a lower risk of cognitive impairment. Our analysis did not show any significant correlation between cognitive impairment and waistline, alcohol drinking, or hemoglobin levels. Thus, some modifiable factors such as physical activity and promotion of increasing HDL levels may be encouraged in Taiwanese older adults.

## Supplementary Information

**Additional file 1: Figure S1.** The selection algorithm of study participants. **Supplementary Table S1.** The distribution of individual SPMSQ score level. **Table S2.** Demographic characteristics of study subjects (including 6366 participants excluded due to missing data). **Table S3.** Comparisons of cognitive impairment group (SPMSQ≧3) and normal cognitive function group (SPMSQ<3) (including 6366 participants excluded due to missing data). **Table S4.** Multivariate logistic regression of factors associated with cognitive impairment(SPMSQ≧3) (including 6366 participants excluded due to missing data).

## Data Availability

The datasets used and/or analyzed during the current study are available from the corresponding author on reasonable request.
