# Supplementary material for: The factors associated with cognitive function among community-dwelling older adults in Taiwan
Source: BMC Geriatr. 2023 Mar 2;23:116. doi: 10.1186/s12877-023-03806-4 (PMC9983251; doi:10.1186/s12877-023-03806-4)
Supplement: Supplementary file 1 — Additional file 1: Figure S1. The selection algorithm of study participants. Supplementary Table S1. The distribution of individual SPMSQ score level. Table S2. Demographic characteristics of study subjects (including 6366 participants excluded due to missing data). Table S3. Comparisons of cognitive impairment group (SPMSQ≧3) and normal cognitive function group (SPMSQ<3) (including 6366 participants excluded due to missing data). Table S4. Multivariate logistic regression of factors associated with cognitive impairment(SPMSQ≧3) (including 6366 participants excluded due to missing data). [file 12877_2023_3806_MOESM1_ESM.docx]

**Figure S1. The selection algorithm of study participants**

**Exclusion(n=48)**

Established Alzheimer disease

**Exclusion (n=6366)**

Missing data

**Enrolled in this study**

**(n=4578)**

**The original cohort of aged health examination participants**

**(n=10992)**

**Supplementary Table S1. The distribution of individual SPMSQ score level**

| **SPMSQ Score** | **Number** | **Percentage (%)** |
| --- | --- | --- |
| **0** | 4053 | 88.5 |
| **1** | 330 | 7.2 |
| **2** | 92 | 2.0 |
| **3** | 41 | 0.9 |
| **4** | 18 | 0.4 |
| **5** | 11 | 0.2 |
| **6** | 6 | 0.1 |
| **7** | 10 | 0.2 |
| **8** | 4 | 0.1 |
| **9** | 9 | 0.2 |
| **10** | 2 | 0.0 |
| **11** | 2 | 0.0 |

**Table S2. Demographic characteristics of study subjects (including 6366 participants excluded due to missing data)**

|  | **All participants(N=10944)** |
| --- | --- |
| **Age (years)** | 73.4 ± 6.2 |
| **Gender (male)** | 4094 (37.4%) |
| **Body height (cm)** | 156.0 ± 8.1 |
| **Body weight (kg)** | 60.0 ± 10.3 |
| **Body mass index (kg/m^2^)** | 24.6 ± 3.6 |
| **Systolic blood pressure (mmHg)** | 136.9 ± 19.9 |
| **Diastolic blood pressure (mmHg)** | 73.3 ± 11.4 |
| **Pulse rate (bpm)** | 74.7 ± 12.0 |
| **Waist circumference (cm)** | 83.8 ± 10.7 |
| **Hypertension (yes)** | 5255 (48.0%) |
| **Diabetes Mellitus (yes)** | 1873 (17.1%) |
| **Hyperlipidemia (yes)** | 1930 (17.6%) |
| **Depression (yes)** | 56(0.5%) |
| **Cardiovascular disease (yes)** | 1940(17.7%) |
| **Osteoporosis (yes)** | 101 (0.9%) |
| **Hyperthyroidism (yes)** | 70 (0.6%) |
| **Smoking in 6 months(yes)** | 632 (5.8%) |
| **Alcohol in 6 months(yes)** | 1566 (14.3%) |
| **Betel nut in 6 months(yes)** | 45 (0.4%) |
| **Exercise in 6 months(yes)** | 5894 (53.9%) |
| **AC sugar (mg/dL)** | 109.0 ± 28.3 |
| **Total protein (g/dL)** | 7.2 ± 0.5 |
| **Albumin (g/dL)** | 4.3± 0.3 |
| **GOT (U/L)** | 25.9 ± 17.7 |
| **GPT (U/L)** | 23.1 ± 19.8 |
| **Creatinine (mg/dL)** | 0.9 ± 0.5 |
| **Total cholesterol (mg/dL)** | 197.3 ± 36.7 |
| **Triglyceride (mg/dL)** | 122.0 ± 73.3 |
| **High density lipoprotein (mg/dL)** | 58.2 ± 16.1 |
| **Uric acid (mg/dL)** | 5.8 ± 1.5 |
| **Hemoglobin (g/dL)** | 13.2 ± 1.4 |
| **SPMSQ score (scores)** | 0.4 ± 1.4 |

The continuous variables were shown as mean ± SD; the categorical variables were shown as percentage.

Abbreviations: SPMSQ, Short Portable Mental Status Questionnaire

**Table S3. Comparisons of cognitive impairment group (SPMSQ≧3) and normal cognitive function group (SPMSQ<3) (including 6366 participants excluded due to missing data)**

|  | **SPMSQ≧3  (N=359)** | **SPMSQ <3  (N=10585)** | ***P* value** |
| --- | --- | --- | --- |
| **Age (years)** | 80.1 ± 7.8 | 73.2 ± 6.0 | **<0.001** |
| **Gender (male)** | 94 (26.2%) | 4000 (37.8%) | **<0.001** |
| **Body height (cm)** | 151.2 ± 7.8 | 156.2 ± 8.1 | **<0.001** |
| **Body weight (kg)** | 55.1 ± 10.2 | 60.1 ± 10.3 | **<0.001** |
| **Body mass index (kg/m^2^)** | 24.0 ± 4.5 | 24.6 ± 3.6 | **0.014** |
| **Systolic blood pressure (mmHg)** | 136.2 ± 22.6 | 136.9 ± 19.8 | 0.533 |
| **Diastolic blood pressure (mmHg)** | 72.4 ± 12.8 | 73.4 ± 11.3 | 0.118 |
| **Pulse rate (bpm)** | 77.7 ± 13.4 | 74.6 ± 11.9 | **<0.001** |
| **Waist circumference (cm)** | 86.1 ± 12.2 | 83.8 ± 10.6 | **<0.001** |
| **Hypertension (yes)** | 180 (50.1%) | 5057 (47.9%) | 0.413 |
| **Diabetes Mellitus (yes)** | 86 (24.0%) | 1787 (16.9%) | **<0.001** |
| **Hyperlipidemia (yes)** | 37 (10.3%) | 1893 (17.9%) | **<0.001** |
| **Depression (yes)** | 3 (0.8%) | 53 (0.5%) | 0.382 |
| **Cardiovascular disease (yes)** | 73 (20.3%) | 1867 (17.6%) | 0.188 |
| **Osteoporosis (yes)** | 2 (0.6%) | 99 (0.9%) | 0.461 |
| **Hyperthyroidism (yes)** | 0 (0%) | 70 (0.7%) | 0.122 |
| **Smoking in 6 months(yes)** | 15 (4.2%) | 617(5.8%) | 0.187 |
| **Alcohol in 6 months(yes)** | 16 (4.5%) | 1550 (14.6%) | **<0.001** |
| **Betel nut in 6 months(yes)** | 1 (0.3%) | 44 (0.4%) | 0.690 |
| **Exercise in 6 months(yes)** | 86 (24.0%) | 5808 (54.9%) | **<0.001** |
| **AC sugar (mg/dL)** | 112.1 ± 39.7 | 108.8 ± 27.9 | 0.120 |
| **Total protein (g/dL)** | 6.9 ± 0.6 | 7.2 ± 0.5 | **<0.001** |
| **Albumin (g/dL)** | 4.1 ± 0.4 | 4.3 ± 0.3 | **<0.001** |
| **GOT (U/L)** | 26.6 ± 21.1 | 25.9 ± 17.6 | 0.520 |
| **GPT (U/L)** | 21.6 ± 27.5 | 23.1 ± 19.5 | 0.165 |
| **Creatinine (mg/dL)** | 1.1 ± 0.6 | 0.9 ± 0.5 | **<0.001** |
| **Total cholesterol (mg/dL)** | 188.9 ± 39.6 | 197.6 ± 36.6 | **<0.001** |
| **Triglyceride (mg/dL)** | 127.6 ± 71.3 | 121.9 ± 73.4 | 0.145 |
| **High density lipoprotein (mg/dL)** | 51.0 ± 13.9 | 58.3 ± 16.1 | **<0.001** |
| **Uric acid (mg/dL)** | 5.9 ± 1.9 | 5.8 ± 1.5 | 0.397 |
| **Hemoglobin (g/dL)** | 12.5 ± 1.6 | 13.2 ± 1.4 | **<0.001** |

The continuous variables were shown as mean ± SD; the categorical variables were shown as percentage. Using chi-squared and t-test; Statistical significance was defined as *P*<0.05.

Abbreviations: SPMSQ, Short Portable Mental Status Questionnaire

**Table S4. Multivariate logistic regression of factors associated with cognitive impairment(SPMSQ≧3) (including 6366 participants excluded due to missing data)**

|  | **Odds Ratio**  **Cognitive decline**  **(SPMSQ≧3 )** | ***P* value** |
| --- | --- | --- |
| **Age (years)** | 1.18 (1.13, 1.22)^*^ | <0.001 |
| **Gender (male)** | 0.48 (0.22, 1.02) | 0.055 |
| **Waist circumference (cm)** | 1.00 (0.98, 1.03) | 0.717 |
| **Diabetes Mellitus (yes)** | 1.52 (0.79, 2.90) | 0.208 |
| **Hyperlipidemia (yes)** | 0.59 (0.27, 1.31) | 0.197 |
| **Alcohol in 6 months(yes)** | 0.75 (0.22, 2.60) | 0.653 |
| **Exercise in 6 months(yes)** | 0.34 (0.25, 0.48)^*^ | <0.001 |
| **Albumin (g/dL)** | 0.26 (0.09, 0.72)^*^ | 0.010 |
| **High density lipoprotein (mg/dL)** | 0.97 (0.95, 0.99)^*^ | 0.005 |
| **Hemoglobin (g/dL)** | 1.06 (0.86, 1.30) | 0.614 |

Odds ratio (OR) and 95% confidence intervals (CIs) are shown after multivariate logistic regression.

*Data are statistically significant (p < 0.05).

Abbreviations: SPMSQ, Short Portable Mental Status Questionnaire
